# Comparative study of the *Rheum tanguticum’s* chemical contents based on spatial distribution characteristics

**DOI:** 10.1371/journal.pone.0278113

**Published:** 2022-11-29

**Authors:** Yafei Guo, Qiang Cao, Mei Guo, Junmei Wang, Renbo Kou, Leilei Ye

**Affiliations:** 1 College of Pharmacy, Gansu University of Chinese Medicine, Lanzhou, China; 2 Key Laboratory for Chemistry and Quality of Traditional Chinese Medicine & Tibetan Medicine of Gansu Provincial Colleges, Lanzhou, China; Universidade Católica Portuguesa Escola Superior de Biotecnologia: Universidade Catolica Portuguesa Escola Superior de Biotecnologia, PORTUGAL

## Abstract

*Rheum tanguticum (R*. *tanguticum)* has been widely used for the treatment of inflammatory diseases in clinical. However, limited research exist on the quality evaluation of various *R*. *tanguticum* locations, which has certain drawbacks. In this study, Fourier-transform infrared spectroscopy (FTIR) and high-performance liquid chromatography (HPLC) were used to comparative study on the chemical contents of *R*. *tanguticum*, to clarify the relationship between the chemical contents and the spatial distribution of *R*. *tanguticum*. First of all, the FTIR spectra of 18 batches of *R*. *tanguticum* were examined. Following the cluster analysis, the FTIR spectra of various production locations differed. To some extent, establishing the double index analysis sequence of common and variation peaks may differentiate distinct production locations of medicinal materials. The HPLC fingerprint of *R*. *tanguticum* was constructed to further explore the link between components and their origin. PCA of common peaks of 18 batches of *R*. *tanguticum* indicated that *R*. *tanguticum* grown in Gannan and Qinghai had a tendency to separate t[2], however this trend was not noticeable. Then, OPLS-DA model was established, and the key differential components of *R*. *tanguticum* produced in Gannan and Qinghai were discovered to be R16, R37, R46, and R47 (Aloe emodin) (*VIP ≥ 1 and P < 0*.*05*). At last, Pearson’s test was used to examine the relationship between longitude, latitude, altitude, and composition. Longitude was significantly positively correlated with R28 and R30 (*P < 0*.*05*), and a very significantly positively correlated with R35, R36, R37, R46, and R47 (*P < 0*.*01*). Latitude was significantly negatively correlated with R34, R35, and R40 (*P < 0*.*05*), and extremely significantly negatively correlated with R28, R30, R36, R37, R46, and R47 (*P < 0*.*01*). Altitude was significantly positive correlation with R36 and R37 (*P < 0*.*01*). The results of our study can provide insights into *R*. *tanguticum* quality control and aid in establishing a natural medication traceability system.

## Introduction

Rhubarb is a traditional Chinese medicine and the most widely used natural medication globally, with primary sources being dried roots and rhizomes. *Rheum tanguticum (R*. *tanguticum)* is one of the main sources of rhubarb. *Rheum tanguticum* Maxim is a Polygonaceae ex Balf plant, which is mostly grown in southern Gansu and southern Qinghai provinces [1]. The primary active constituents of rhubarb are anthraquinone, double anthrone, and tannin. Its primary pharmacological effects include inducing constipation and anti-inflammatory, diuretic, antibacterial, and antitumor properties [2–4]. Anti- inflammatory is one of the most important pharmacological effects of rhubarb. Rhubarb has been also used to treat inflammation-related diseases in clinical, such as acute pancreatitis, acute cholecystitis, appendicitis, ulcerative colitis and so on [5–9]. The administration of rhubarb extract reduced the expression of key inflammatory genes (TLR4, MCP-1, TNF-α, IL-6), macrophage-related markers (F4/80, CD68, CD11c) and oxidative markers (TLR4, NADPH oxidase). Rhubarb can eliminate inflammatory mediators from tissue and plasma and drastically reduce tumor necrosis factor (TNF), interleukin, and endotoxin levels in the serum of patients suffering from or successfully treated with severe pancreatitis by blocking the production of the inflammatory factor HMGB-1 [10]. Rhubarb aqueous extract not only inhibit the inflammatory response induced by TNF-α and the augmentation or rise of adhesion molecules in human umbilical vein endothelial cells (HUVECs), but also reduce adhesion between U937 cells and HUVECs [11]. The rhubarb extract modified host antimicrobial peptide production and gut homeostasis and was associated with profound changes in gut microbial composition [12,13]. Previous studies showed that rhubarb components, such as emodin, rhein, chrysophanol, lindleyin, and isolotus anthocyanins, have anti-inflammatory properties [11,14,15]. Emodin inhibits lipopolysaccharide (LPS)-induced apoptosis and inflammation by increasing the expression of the TUG1 (Long non-coding RNA) in cartilage-derived ATDC5 cells [16]. Emodin and Rhein exhibit potent anti-inflammatory actions in LPS-induced Raw 264.7 cells [17,18]. Chrysophanol may ameliorate LPS-induced inflammation in mice, minimize pathological harm to the liver and lung tissues, and lower blood TNF-α levels [19]. Derived from rhubarb, lindleyin and isolotus palmoside have anti-inflammatory and analgesic properties [20]. For the study of the anti-inflammatory mechanism of rhubarb, most of them focus on the study of components (such as: emodin, rhein) [21,22]. Several studies showed that the inflammatory effect of emodin was involved in the inactivation of NF-κB, an essential regulator of inflammatory processes, and PPARγ-dependent pathway was possibly involved in the NF-κB inhibitive effect of emodin [23,24]. Rhein significantly decreased IL-1β secretion via NLRP3 inflammasomes by disturbing their assembly in macrophages. Rhein also activated the Nrf2-HO1-NQO1 pathway and inhibited expression of Nox2 subunits and translocation to regulate redox balance. Moreover, rhein attenuated inflammatory responses by mediating macrophage polarization from M1 to M2 phenotype. NF-κB, AP-1, and MAPK signalling were also involved in improving inflammatory conditions by rhein [25–27]. With the development and progress of natural medication, quality control has been the most crucial factor in current research [20]. The quality of rhubarb in the market is variable because of mixed varietals, varying processing processes, and manufacturing locales [28]. Numerous studies have been conducted on the quality control of rhubarb to maximize the impact of natural medications; however, those on the variations in *R*. *tanguticum* from different production locations are limited [29–31]. This is not conducive to the clinical application of *R*. *tanguticum* from different origins.

To better manage the internal quality of natural drugs, modern analytical methods, such as spectroscopy and chromatography, have been widely utilized in quality control to compensate for the weakness of conventional morphological and anatomical identification of the source of natural pharmaceuticals. These sophisticated analytical approaches not only objectively represent internal quality, but also compensate for the lack of personal expertise. Some authors have recently described plant drug control analytical approaches, such as chromatography, spectrum, DNA barcoding, and other analytical methods [32–35]. Spectral and chromatographic approaches are mostly utilized in categorizing and identifying original plants as well as in evaluating quality and identifying the origin. These two approaches can explain some additional chemical information simultaneously, and the resulting fingerprint has become an established method for evaluating quality consistency. Fourier-transform infrared spectroscopy (FTIR) and high-performance liquid chromatography (HPLC) are two such techniques. FTIR has been widely used to determine the provenance of food and natural pharmaceuticals as well as to forecast the quantity of index components [36,37]. Many agencies, including the World Health Organization, Food and Drug Administration, and State Food and Drug Administration, have acknowledged HPLC fingerprint [38]. These two approaches, in conjunction with chemometrics, have been effectively used for the quality control and origin identification of a wide range of natural pharmaceuticals. Simple and quick measurement methods bypass the time-consuming pretreatment procedure for quality analysis and do not require a significant number of chemical reagents.

In this study, the FTIR spectra of 18 batches of *R*. *tanguticum* were examined by FTIR. Cluster analysis and the double index sequence method were used for distinguish *R*. *tanguticum* regions. The HPLC fingerprint of *R*. *tanguticum* was constructed by HPLC. And PCA, OPLS-DA was used for study the differential components of *R*. *tanguticum* in Gannan and Qinghai. At last, pearson correlation test was used for preliminarily clarified the relationship between chemical contents of *R*. *tanguticum* and longitude, latitude, altitude. The results of this study could encourage the growth of *R*. *tanguticum* business and to assist in implementing a natural medication traceability system.

## Materials and methods

### Herbal medicine

Yuwei Gan, Agricultural Technology Extension Researcher at the Gannan Science and Technology Development and Exchange Center, confirmed the authenticity of the *R*. *tanguticum* sample. For more information, refer to Table 1.

**Table 1:** Rheum tanguticum sample information.

label	Collecting Time	Source	Longitude/°E	Latitude/°N	Altitude/m
**1**	Autumn, 2017	Hezuo	102.91	35.00	3136
**2**	Spring, 2019	Hezuo	102.91	35.00	3136
**3**	Autumn, 2019	Hezuo	102.91	35.00	3136
**4**	Autumn, 2018	Hezuo	102.91	35.00	3136
**5**	Autumn, 2018	Hezuo	102.91	35.00	3136
**6**	Autumn, 2018	Hezuo	102.91	35.00	3136
**7**	Autumn, 2018	Hezuo	102.91	35.00	3136
**8**	Autumn, 2017	Hezuo	102.91	35.00	3136
**9**	Autumn, 2019	Xiahe, Hezuo	102.52	35.20	3471
**10**	Autumn, 2019	Lintan, Hezuo	103.35	34.69	2867
**11**	Autumn, 2019	Qinghai	101.77	36.65	2261
**12**	Autumn, 2019	Qinghai	101.77	36.65	2261
**13**	Autumn, 2018	Qinghai	101.77	36.65	2261
**14**	Autumn, 2018	Qinghai	101.77	36.65	2261
**15**	Autumn, 2019	Huangzhong, Qinghai	101.57	36.50	2600
**16**	Autumn, 2018	Huangzhong, Qinghai	101.57	36.50	2600
**17**	Autumn, 2018	Datong, Qinghai	101.69	36.93	3451
**18**	Autumn, 2017	Guide, Qinghai	101.44	36.07	2200

### FTIR study

#### Instrument parameter setting

A Nicolet 6700 FT-IR spectrometer (Thermo Fisher, America) with a silicon carbide rod light source, DTGs detector, 4 cm^-1^ resolution, 30 scanning accumulation, detection in the spectral range of 4000–400 cm^-1^, room temperature, and the interference of H_2_O and CO_2_ was deducted.

#### Sample preparation

*R*. *tanguticum* was crushed and sieved through a 100 mesh sieve (Huafeng Hardware Instrument Co., Ltd., Shaoxing, China). Ultrasonic extraction of 1.00 g of powder with 50 mL methanol (AR, Lot No.: 20200825, Tianjin Fuyu Fine Chemical Co., Ltd., Tianjin, China) was performed for 1 h (20 kHz, 30±5 °C). Then, suction filtration was performed and the methanol was evaporated to obtain the *R*. *tanguticum* methanol extract, which was labeled M1–M18.

Approximately 1 mg of *R*. *tanguticum* methanol-extracted powder was mixed with 100 mg of KBr (for IR, Lot No.: 20200715, Damao Chemical Reagent Factory, Tianjin, China) and compressed into a tablet. KBr was used as a blank, and infrared spectrum data for both samples were collected.

#### Infrared spectrum data analysis

The OMNIC 8.2 software and Origin software (version 9.0) were used for automated baseline correction, smoothing, and ordinate normalization of infrared spectrum findings.

### Double index sequence analysis of common peak rate and variation peak rate


$$P=\frac{Ng}{N}\times 100\%$$ (1)

$$Pa=\frac{Na}{Ng}\times 100\%$$ (2)

$$Pb=\frac{Nb}{Ng}\times 100\%$$ (3)

Note: P is the common peak rate of the two samples; N is the number of independent peaks in the two samples; Ng is the common peak number of the two samples; Pa is the variation peak rate of the sample; Na is the variation peak rate of the sample; Pb is the variation peak rate of sample B; Nb is the variation peak number of sample B, which conforms to N = Ng + Na + Nb.

The common peak rate and variation peak rate of the infrared spectrum between M1 and M18 were determined using (1)–(3), and the Rhubarb double index analysis sequence was created. The sequence has great identification ability and can accurately estimate the genetic distance between all samples in the 2+n-dimensional (n = number of samples) space [39]. Ma: Mb (P; Pa, Pb) is the double index analysis sequence of the common peak rate and variation peak rate, which implies that with Ma as the control medication, the common peak rate of Mb is P, the variation peak rate of Ma is Pa, and the variation peak rate of Mb is Pb.

### HPLC study

#### Chromatographic conditions

The Agilent 1260 High performance liquid chromatography (Agilent Technologies, USA), with an Agilent TC-C18(2) chromatographic column (4.6×250 mm, 5 μm) was utilized. The flow rate was 1 mL·min^-1^, column temperature was 30 °C, detection wavelength was 280 nm, and injection volume was 5 μL. The mobile phase comprised methanol (A) and –0.1% formic acid in water (B). The gradient elution procedure was: 0–6 min, 10%–30% A; 6–13 min, 30%–33% A; 13–21 min, 33%–37% A; 21–25 min, 37%–40% A; 25–35 min, 40%–45% A; 35–45 min, 45%–48% A; 45–49 min, 48%–52% A; 49–54 min, 52%–60% A; 54–64 min, 60%–95% A; 64–74 min, 95%–10% A; 74–78 min, 10% A.

#### Sample preparation

Ultrasonic extraction was performed on 1.00 g rhubarb powder and 50 mL methanol for 1 h (20 kHz, 30±5 °C). The sample was filtered using 0.45 μm microporous membrane filtration. The filtered sample was marked as S1–S18.

The single standard and mixed standard solutions were prepared with methanol at a concentration of 1 mg/mL for each pharmacopoeia standard (purity ≥ 98%) of emodin (Lot No. 1205A0210), rhein (Lot No. 109C021), chrysophanol (Lot No. 523D025), aloe emodin (Lot No. 531B024), emodin methyl ether (Lot No. 718C023), catechin (Lot No. 1125H021), gallic acid (Lot No. 725G028), and sennoside A (Lot No. AF9111318) and sennoside B (Lot No. AF9111319).

#### Establish fingerprint

The S1–S18 chromatographic data was imported into the "Similarity evaluation system for chromatographic fingerprint of TCM (Version 2012),” for which the *R*. *tanguticum* fingerprint was created. Then, the similarity of S1–S18 was analyzed.

#### Analysis of chemometrics methods

The spectrum of S1–S18 chromatographic data was imported into the SIMCA software. The missing value was replaced with half of the minimum value, and the component was eliminated if the missing value reached 20%. The HPLC data matrix was statistically analyzed using PCA. The data were separated into two groups based on their origin, and OPLS-DA was performed to compare the groups.

## Results and analysis

### The results from the FTIR study

#### Similarity analysis results

**Fig 1:**
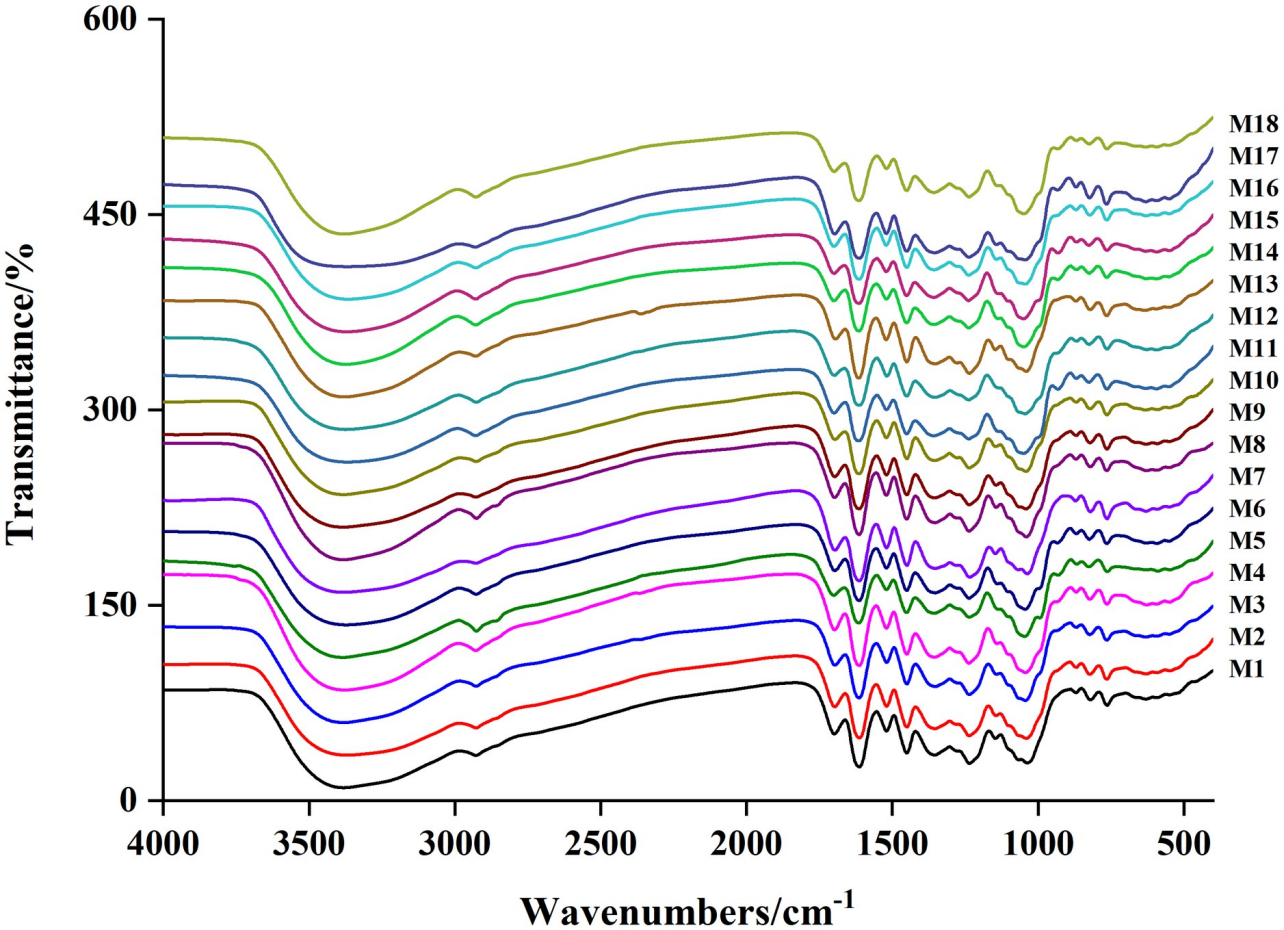
The FTIR spectra of M1 ~ M18.

The FTIR spectra of M1–M18 are shown in Fig 1 and exhibit certain similarities. Absorption peaks were observed at 400–1800 cm^-1^ and 2700–3800 cm^-1^.

The spectra were analyzed for similarity; the results are presented in Table 2. The similarity of the FTIR spectra ranged from 0.863 to 0.988, with a high overall similarity, demonstrating that the composition and content of *R*. *tanguticum* were relatively stable. Regarding sample similarity analysis, in M1–M10, 97.78 percent were greater than 0.9 and 96.43% of the samples in M11–M18 had similarity greater than 0.9. The similarity between M1–M10 and M11–M18 was greater than 0.9, accounting for 92.50%, demonstrating a high similarity between the M1–M10 and M11–M18 groups. The similarity between M1–M10 and M11–M18 was slightly lower.

**Table 2:** Similarity analysis of FTIR spectrum of M1~M18.

**Sample**	**M1**	**M2**	**M3**	**M4**	**M5**	**M6**	**M7**	**M8**	**M9**	**M10**	**M11**	**M12**	**M13**	**M14**	**M15**	**M16**	**M17**	**M18**
**M1**	1.000																	
**M2**	0.929	1.000																
**M3**	0.969	0.967	1.000															
**M4**	0.977	0.930	0.957	1.000														
**M5**	0.991	0.927	0.968	0.981	1.000													
**M6**	0.919	0.938	0.966	0.893	0.915	1.000												
**M7**	0.972	0.950	0.972	0.960	0.969	0.918	1.000											
**M8**	0.976	0.956	0.969	0.988	0.979	0.907	0.973	1.000										
**M9**	0.945	0.950	0.982	0.922	0.940	0.970	0.954	0.936	1.000									
**M10**	0.951	0.974	0.989	0.932	0.945	0.974	0.963	0.950	0.985	1.000								
**M11**	0.954	0.948	0.981	0.923	0.948	0.975	0.953	0.933	0.982	0.982	1.000							
**M12**	0.892	0.955	0.959	0.863	0.884	0.973	0.917	0.890	0.974	0.981	0.969	1.000						
**M13**	0.970	0.971	0.982	0.966	0.971	0.946	0.974	0.977	0.966	0.974	0.972	0.936	1.000					
**M14**	0.967	0.913	0.944	0.961	0.960	0.890	0.951	0.962	0.924	0.929	0.930	0.867	0.964	1.000				
**M15**	0.970	0.968	0.987	0.951	0.969	0.959	0.975	0.964	0.974	0.983	0.983	0.954	0.989	0.947	1.000			
**M16**	0.963	0.962	0.985	0.944	0.957	0.975	0.961	0.954	0.983	0.987	0.984	0.965	0.982	0.947	0.985	1.000		
**M17**	0.941	0.973	0.983	0.920	0.936	0.975	0.957	0.940	0.982	0.992	0.984	0.983	0.972	0.920	0.984	0.985	1.000	
**M18**	0.927	0.942	0.965	0.887	0.915	0.966	0.933	0.906	0.969	0.977	0.973	0.974	0.940	0.892	0.964	0.969	0.982	1.000

The similarity of rhubarb methanol extract from the same origin was significant, whereas that of rhubarb from other sources was somewhat different. The rhubarb-producing locations can be differentiated using infrared chromatography.

#### Confirmation of common peaks and peak attribution

If the greatest difference in the wave number of a group of absorption peaks was smaller than the difference in the average wave number of its surrounding groups of peaks, the group of peaks was defined as a group of common peaks.

According to the absorption peak data reported by the OMNIC software, 17 peaks were found in the alcohol extract using standard peak identification methods. The wavenumber and common FTIR absorption peaks were listed (Table 3). Common peaks in the distinctive region were assigned as Table 4.

**Table 3:** Common peaks of M1~M18 FTIR absorption.

**Sample**	**Wave number/cm** ^**-1**^								
**M1**	3380.70	2927.14	1697.90	1609.27	1513.93	1449.18	1354.62	1238.54	—
**M2**	3369.65	2929.21	1697.46	1609.02	1514.56	1448.76	1355.84	1237.49	1142.48
**M3**	3365.17	2927.10	1698.14	1609.14	1516.28	1448.93	1355.10	1238.36	1142.77
**M4**	3372.86	2928.06	1697.77	1609.98	1513.94	1449.07	1358.24	1238.85	—
**M5**	3373.23	2927.81	1700.74	1610.45	1514.33	1449.34	1354.00	1239.39	—
**M6**	3377.44	2928.13	1696.30	1611.60	1518.16	1448.93	1356.21	1236.71	1143.82
**M7**	3373.34	2925.65	1698.39	1608.92	1513.96	1448.88	1355.33	1239.76	—
**M8**	3369.39	2928.44	1697.80	1609.48	1513.64	1449.08	1357.40	1238.86	—
**M9**	3381.26	2926.57	1696.72	1609.89	1514.66	1448.86	1353.83	1237.44	1142.66
**M10**	3384.23	2926.82	1697.2	1609.02	1514.14	1448.9	1355.01	1237.69	1142.93
**M11**	3383.08	2924.68	1697.66	1610.43	1515.44	1448.74	1352.77	1238.20	1142.69
**M12**	3380.62	—	1696.67	1609.32	1513.83	1448.65	1353.16	1235.59	1143.66
**M13**	3372.38	2927.02	1697.51	1610.12	1514.12	1449.00	1355.88	1237.88	1141.40
**M14**	3385.58	2925.70	1701.26	1610.35	1514.26	1449.86	1359.77	1238.85	—
**M15**	3374.93	2927.39	1698.17	1609.31	1514	1448.81	1359.02	1238.53	1141.64
**M16**	3377.28	2926.61	1697.03	1611.27	1512.78	1448.79	1354.26	1236.87	1142.65
**M17**	3376.22	2926.82	1697.52	1609.02	1513.33	1448.57	1355.31	1237.06	1143.23
**M18**	3381.82	2926.51	1697.71	1609.27	1513.68	1448.78	1356.17	1236.50	1143.94
**Average**	3376.62	2927.04	1697.89	1609.77	1514.39	1448.95	1355.66	1237.92	1142.82
**Maximum difference**	20.41	4.53	4.96	2.68	5.38	1.29	7.00	4.17	2.54
**Sample**	**Wave number/cm** ^**-1**^								
**M1**	1048.35	—	869.52	825.15	766.81	629.04	—	—	
**M2**	1044.86	944.03	869.54	823.00	767.14	627.93	—	547.63	
**M3**	1044.46	—	869.47	818.49	766.67	630.74	—	—	
**M4**	1048.82	926.47	870.92	822.44	767.20	—	594.23	—	
**M5**	1048.76	—	870.64	819.03	767.21	—	598.34	—	
**M6**	1038.13	—	868.09	821.76	767.35	634.63	—	—	
**M7**	1045.11	—	869.04	826.91	766.31	—	590.36	—	
**M8**	1049.24	926.40	868.71	826.88	767.18	—	593.47	—	
**M9**	1037.93	—	871.05	818.49	766.24	630.89	—	—	
**M10**	1038.07	—	869.30	818.37	766.74	628.60	—	—	
**M11**	1038.08	—	869.10	819.10	766.84	627.45	—	—	
**M12**	1035.60	—	870.04	818.61	766.29	629.53	—	—	
**M13**	1044.86	—	868.77	825.29	766.59	—	596.19	—	
**M14**	1045.96	—	867.50	826.28	765.96	—	584.16	—	
**M15**	1041.91	—	869.19	817.62	766.28	628.76	—	547.37	
**M16**	1040.83	—	868.43	822.38	766.47	625.63	—	—	
**M17**	1038.24	—	869.64	818.32	766.45	629.04	—	—	
**M18**	1035.73	—	869.48	817.30	765.36	628.65	—	—	
**Average**	1042.50	932.30	869.36	821.41	766.67	629.24	592.79	547.50	
**Maximum difference**	13.64	17.63	3.55	9.61	1.99	9.00	14.18	0.26	

"-" means that the sample has no this wave number.

**Table 4:** Vibrational modes of functional groups in common peaks (distinctive region).

Wave number/cm^-1^	Peak intensity	Vibration mode
3415.27–3359.21	wide and strong peak	polymer ν (O-H), amide ν (N-H)
2932.79–2924.68	acromion	methylene νas(C-H)
1701.26–1696.30	moderately strong peak	ν (C = O)
1626.70–1608.92	medium strong peak	amide β (N-H)
1520.35–1512.78	weak peak	amide β (N-H), aromatic hydrocarbon, and benzene ring skeleton vibration ν (C = C)
1449.86–1446.70	medium strong peak	methyl δ (C-H), methylene δ (C-H), β (O-H), aromatic hydrocarbon, and benzene ring skeleton vibration ν (C = C)
1367.46–1316.93	medium strong peak	methyl β (C-H)
1243.07–1235.59	moderately strong peak	δ (O-H), ν (C-N), and ν (C-O-C)
1146.30–1141.00	medium strong peak	ν (C-N) of lipid compounds ν (C-O-C)
1053.72–1033.93	strong peak	δ (C-O)

*R*. *tanguticum* was mostly composed of anthraquinones, polysaccharides, and tannins. The FTIR analysis results were consistent with the composition research findings, and the methanol extract included the majority of the *R*. *tanguticum* components.

#### Cluster analysis

**Fig 2:**
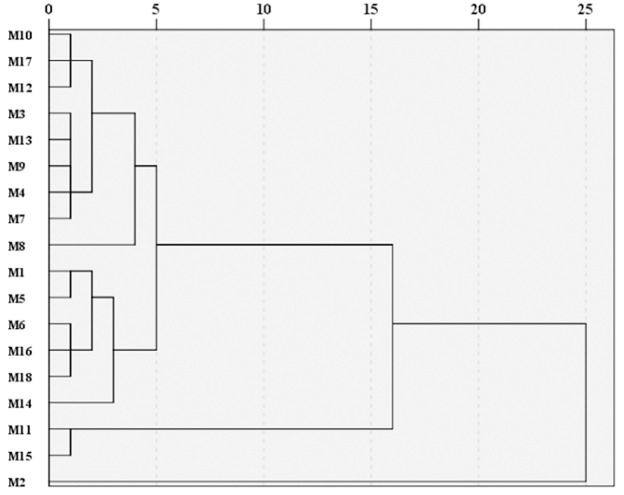
FTIR cluster analysis diagram of M1 ~ M18.

SPSS 26 software was used to perform systematic cluster analysis of M1–M18. The interval was the square Euclidean distance, and the cluster technique was the intergroup connection method. Cluster analysis was performed on 18 batches of FTIR spectra (Fig 2). When the Euclidean Distance was 10, it was separated into three groups: M10M17M12M3M13M9M4M7M8M1M5M6M16M18M14, M11M15, and M2. This might be owing to the cumulative impact of geographical location, soil, and weather. The chemical content of rhubarb varied depending on the location of its growth.

#### The results of the double index sequence analysis

Basic grouping was employed in the M1–M18 sequence. According to the common peak rate, the most comparable sample pairs and groups could be sorted into the Tables 5–8.

**Table 5:** The sequence with the same absorption peak (Group A).

Control Sample	Comparison Sample	(P; Pa, Pb)	Control Sample	Comparison Sample	(P; Pa, Pb)
M3	M6M9M10M11M16M17M18	(1.0000; 0.0000, 0.0000)	M4	M8	(1.0000; 0.0000, 0.0000)
M5	M7M14	(1.0000; 0.0000, 0.0000)	M6	M3M9M10M11M16M17M18	(1.0000; 0.0000, 0.0000)
M7	M5M14	(1.0000; 0.0000, 0.0000)	M8	M4	(1.0000; 0.0000, 0.0000)
M9	M3M6M10M11M16M17M18	(1.0000; 0.0000, 0.0000)	M10	M3M6M9M11M16M17M18	(1.0000; 0.0000, 0.0000)
M11	M3M6M9M10M16M17M18	(1.0000; 0.0000, 0.0000)	M14	M5M7	(1.0000; 0.0000, 0.0000)
M16	M3M6M9M10M11M17M18	(1.0000; 0.0000, 0.0000)	M17	M3M6M9M10M11M16M18	(1.0000; 0.0000, 0.0000)
M18	M3M6M9M10M11M16M17	(1.0000; 0.0000, 0.0000)			

**Table 6:** The sequence with high common peak rate and low variation peak rate (Group B).

Control Sample	Comparison Sample	(P; Pa, Pb)	Control Sample	Comparison Sample	(P; Pa, Pb)
M1	M3M6M9M10M11M16M17M18	(0.9286; 0.0000, 0.0769)	M1	M5M7M12M14	(0.8571; 0.0833, 0.0833)
M2	M15	(0.9375; 0.0667, 0.0000)	M2	M1M12M13	(0.8125; 0.2308, 0.0000)
M3	M15	(0.9333; 0.0000, 0.0714)	M3	M1M12	(0.9286; 0.0769, 0.0000)
M3	M13	(0.8667; 0.0769, 0.0769)	M4	M5M7M14	(0.9286; 0.0769, 0.0000)
M4	M13	(0.8125; 0.0769, 0.0769)	M5	M4M8M13	(0.9286; 0.0000, 0.0769)
M5	M1	(0.8571; 0.0833, 0.0833)	M6	M15	(0.9333; 0.0000, 0.0714)
M6	M1M12	(0.9286; 0.0769, 0.0000)	M6	M13	(0.8667; 0.0769, 0.0769)
M7	M4M8M13	(0.9286; 0.0000, 0.0769)	M7	M1	(0.8571; 0.0833, 0.0833)
M8	M5M7M14	(0.9286; 0.0769, 0.0000)	M8	M13	(0.8667; 0.0769, 0.0769)
M9	M15	(0.9333; 0.0000, 0.0714)	M9	M1M12	(0.9286; 0.0769, 0.0000)
M9	M13	(0.8667; 0.0769, 0.0769)	M10	M15	(0.9333; 0.0000, 0.0714)
M10	M1M12	(0.9286; 0.0769, 0.0000)	M10	M13	(0.8667; 0.0769, 0.0769)
M11	M15	(0.9333; 0.0000, 0.0714)	M11	M1M12	(0.9286; 0.0769, 0.0000)
M11	M13	(0.8667; 0.0769, 0.0769)	M12	M3M6M9M10 M11M16M17M18	(0.9286; 0.0000, 0.0769)
M12	M1	(0.8571; 0.0833, 0.0833)	M13	M5M7M14	(0.9286; 0.0769, 0.0000)
M13	M3M6M8M9M10 M11M16M17M18	(0.8667; 0.0769, 0.0769)	M13	M4	(0.8125; 0.0769, 0.0769)
M14	M4M8M13	(0.9286; 0.0000, 0.0769)	M14	M1	(0.8571; 0.0833, 0.0833)
M15	M2	(0.9375; 0.0000, 0.0667)	M15	M3M6M9M10 M11M16M17M18	(0.9333; 0.0714, 0.0000)
M16	M15	(0.9333; 0.0000, 0.0714)	M16	M1M12	(0.9286; 0.0769, 0.0000)
M16	M13	(0.8667; 0.0769, 0.0769)	M17	M15	(0.9333; 0.0000, 0.0714)
M17	M1M12	(0.9286; 0.0769, 0.0000)	M17	M13	(0.8667; 0.0769, 0.0769)
M18	M15	(0.9333; 0.0000, 0.0714)	M18	M1M12	(0.9286; 0.0769, 0.0000)
M18	M13	(0.8667; 0.0769, 0.0769)			

**Table 7:** The sequence with high common peak rate and high variation peak rate (Group C).

Control Sample	Comparison Sample	(P; Pa, Pb)	Control Sample	Comparison Sample	(P; Pa, Pb)
M1	M15	(0.8667; 0.0000, 0.1538)	M1	M2	(0.8125; 0.000, 0.2308)
M1	M4M8M13	(0.8000; 0.0833, 0.1667)	M2	M3M6M9M10 M11M16M17M18	(0.8750; 0.1429, 0.0000)
M3	M2	(0.8750; 0.0000, 0.1429)	M3	M5M7M14	(0.8000; 0.1667, 0.0833)
M4	M1	(0.8000; 0.1667, 0.0833)	M5	M3M6M9M10 M11M16M17M18	(0.8000; 0.0833, 0.1667)
M6	M2	(0.8750; 0.0000, 0.1429)	M6	M5M7M14	(0.8000; 0.1667, 0.0833)
M7	M3M6M9M10M11M16M17M18	(0.8000; 0.0833, 0.1667)	M8	M1	(0.8000; 0.1667, 0.0833)
M9	M2	(0.8750; 0.0000, 0.1429)	M9	M5M7M14	(0.8000; 0.1667, 0.0833)
M10	M2	(0.8750; 0.0000, 0.1429)	M10	M5M7M14	(0.8000; 0.1667, 0.0833)
M11	M2	(0.8750; 0.0000, 0.1429)	M11	M5M7M14	(0.8000; 0.1667, 0.0833)
M12	M15	(0.8667; 0.0000, 0.1538)	M12	M2	(0.8125; 0.0000, 0.2308)
M12	M13	(0.8000; 0.0833, 0.1667)	M13	M15	(0.8125; 0.0769, 0.1538)
M13	M2	(0.8125; 0.0000, 0.2308)	M13	M1M12	(0.8000; 0.1667, 0.0833)
M14	M3M6M9M10M11M16M17M18	(0.8000; 0.0833, 0.1667)	M15	M1M12	(0.8667; 0.1538, 0.0000)
M15	M13	(0.8125; 0.1538, 0.0769)	M16	M2	(0.8750; 0.0000, 0.1429)
M16	M5M7M14	(0.8000; 0.1667, 0.0833)	M17	M2	(0.8750; 0.0000, 0.1429)
M17	M5M7M14	(0.8000; 0.1667, 0.0833)	M18	M2	(0.8750; 0.0000, 0.1429)
M18	M5M7M14	(0.8000; 0.1667, 0.0833)			

**Table 8:** The sequence with low common peak rate and high variation peak rate (Group D).

Control Sample	Comparison Sample	(P; Pa, Pb)	Control Sample	Comparison Sample	(P; Pa, Pb)
M2	M4M8	(0.7674; 0.2308, 0.0769)	M2	M5M7M14	(0.7059; 0.3333, 0.0833)
M3	M4M8	(0.7500; 0.1667, 0.1667)	M4	M2	(0.7674; 0.0769, 0.2308)
M4	M3M6M9M10M11M16M17M18	(0.7500; 0.1667, 0.1667)	M4	M15	(0.7059; 0.1667, 0.1667)
M4	M12	(0.6875; 0.2727, 0.0909)	M5	M15	(0.7500; 0.0833, 0.2500)
M5	M12	(0.7333; 0.1818, 0.1818)	M5	M2	(0.7059; 0.0833, 0.3333)
M5	M2	(0.7059; 0.0833, 0.3333)	M6	M4M8	(0.7500; 0.1667, 0.1667)
M7	M15	(0.7500; 0.0833, 0.2500)	M7	M12	(0.7333; 0.1818, 0.1818)
M7	M2	(0.7059; 0.0833, 0.3333)	M8	M2	(0.7674; 0.0769, 0.2308)
M8	M3M6M9M10M11M16M17M18	(0.7500; 0.1667, 0.1667)	M8	M15	(0.7059; 0.1667, 0.2500)
M8	M12	(0.6875; 0.2727, 0.1818)	M9	M4M8	(0.7500; 0.1667, 0.1667)
M10	M4M8	(0.7500; 0.1667, 0.1667)	M11	M4M8	(0.7500; 0.1667, 0.1667)
M12	M5M7	(0.7333; 0.1818, 0.1818)	M12	M8	(0.6875; 0.1818, 0.2727)
M12	M14	(0.6875; 0.1818, 0.1818)	M12	M4	(0.6875; 0.0909, 0.2727)
M14	M15	(0.7500; 0.0833, 0.2500)	M14	M2	(0.7059; 0.0833, 0.3333)
M14	M12	(0.6875; 0.1818, 0.1818)	M15	M5M7	(0.7500; 0.2500, 0.0833)
M15	M14	(0.7500; 0.2500, 0.0833)	M15	M8	(0.7059; 0.2500, 0.1667)
M15	M4	(0.7059; 0.1667, 0.1667)	M16	M4M8	(0.7500; 0.1667, 0.1667)
M17	M4M8	(0.7500; 0.1667, 0.1667)	M18	M4M8	(0.7500; 0.1667, 0.1667)

The common peak rate and variation peak rate of the sequence were the same in group A, and the absorption peak corresponded completely. The sequence common peak rate was high in group B, whereas its variation peak rate was low. The sequence common peak rate and variation peak rate were higher in Group C. The common peak rate of the sequence was lower in Group D, whereas its variation peak rate was higher.

Double index analysis and sequence analysis of the methanol extract of rhubarb revealed that different results of *R*. *tanguticum* from different producing areas. However, the methanol extract can eliminate some differences caused by environmental factors. For example, Hezuo, Hezuo, Xiahe of Hezuo, Lintan of Hezuo, Qinghai, Huangzhong of Qinghai, Datong of Qinghai, and Guide of Qinghai are the corresponding producing areas of sequence M3:M6M9M10M11M16M17M18. Nonetheless, the sequence resulting from double index analysis showed that the common peak rate was 100%, with no variation peak. If this method was used, then the samples were classified into one category.

According to the results of the above analysis, environmental factors such as origin, altitude, longitude, latitude, and climatic conditions could affect the common peak rate and variation peak rate of *R*. *tanguticum*, but the specific impact could be determined. The components of *R*. *tanguticum* were determined using HPLC, and the effects of altitude, longitude, and latitude on these components were studied to further analyze the influence of the environment on the quality of *R*. *tanguticum*.

### The result of HPLC study

#### Chromatography of pharmacopoeia standards

**Fig 3:**
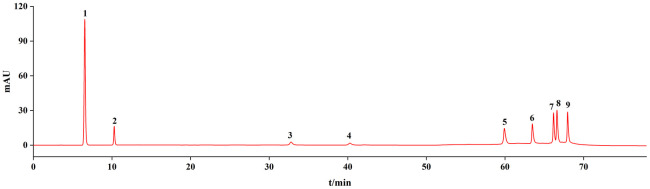
Chromatogram of pharmacopoeia standards. 1: Gallic acid, 2: (+) -catechin, 3: Sennoside B, 4: Sennoside A, 5: Aloe emodin, 6: Rhein, 7: Emodin, 8: Chrysophanol, 9: Emodin methyl ether.

The chromatographic peaks of the mixed pharmacopoeia standards are shown in Fig 3. The chromatographic peaks of the nine components were identified using the chromatographic peak of a single pharmacopoeia standard.

#### Establish rhubarb fingerprint

**Fig 4:**
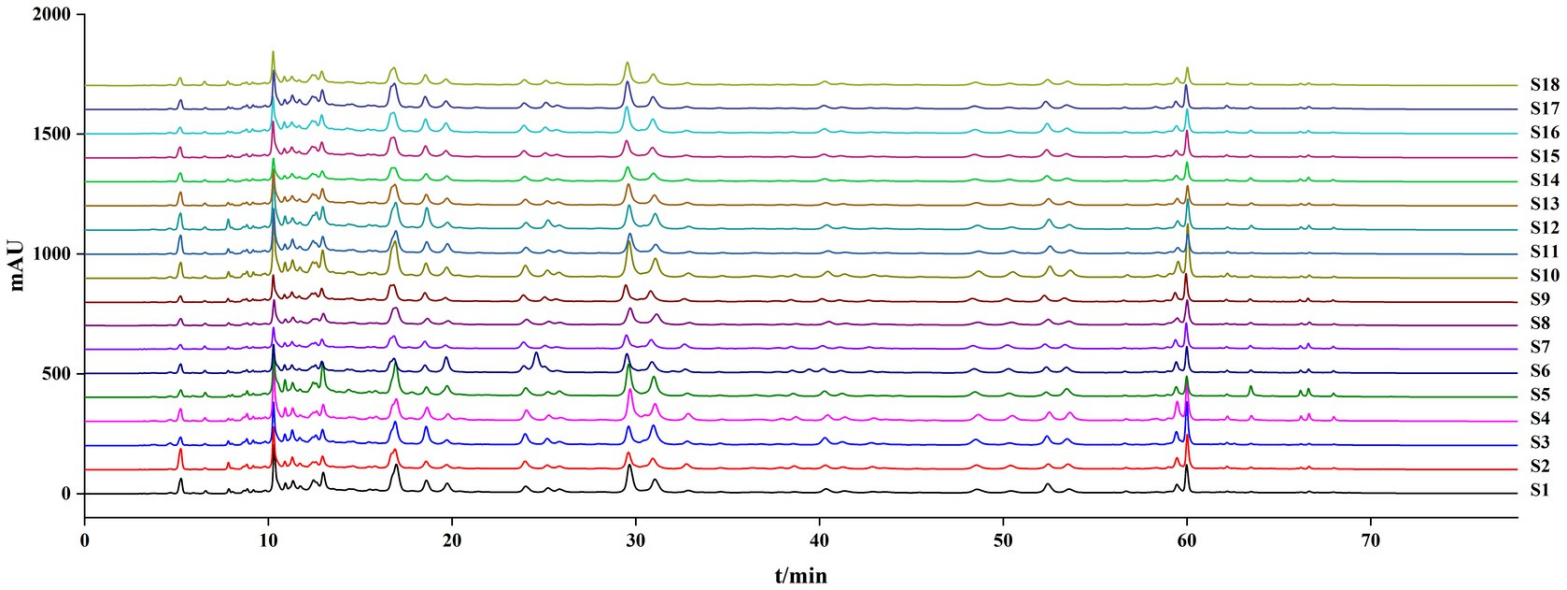
The chromatograms of S1 ~ S18.

**Fig 5:**
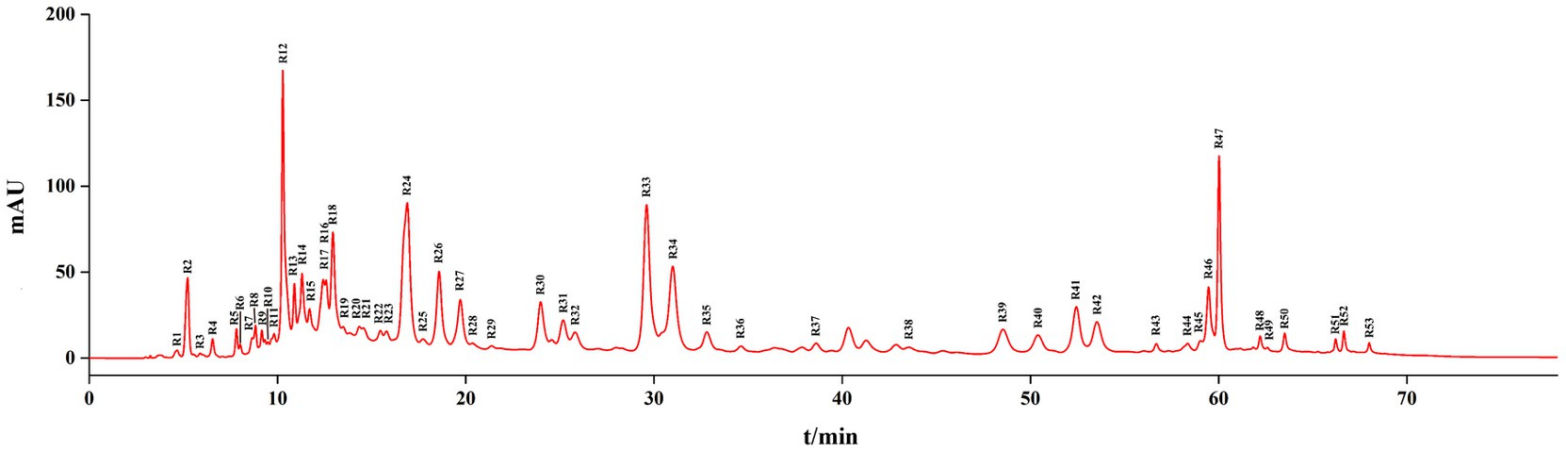
The chromatogram of S1 ~ S18 (common peak mode). R4: Gallic acid, R12: (+)—catechin, R35: Sennoside B, R47: Aloe emodin, R50: Rhein, R51: Emodin, R52: Chrysophanol, R53: Emodin methyl ether.

HPLC was used to acquire chromatograms of S1–S18 (Fig 4). The S1–S18 spectra were imported into the "Similarity evaluation system for chromatographic fingerprint of TCM (version 2012)" and S1 was set as the control spectrum for chromatographic peak matching to obtain the *R*. *tanguticum* common peak pattern spectrum (Fig 5). A total of 53 peaks were observed. Eight chromatographic peaks were identified after the retention time and compared with the chromatogram of the reference material.

The fingerprints of the 18 batches of Rheum tanguticum were nearly identical. The similarity of each batch to the standard fingerprint was 0.909, 0.861, 0.888, 0.895, 0.879, 0.620, 0.671, 0.899, 0.917, 0.909, 0.918, 0.914, 0.853, 0.689, 0.668, 0.779, 0.734, and 0.734, all of which are between 0.62 and 0.92.

#### PCA analysis results

**Fig 6:**
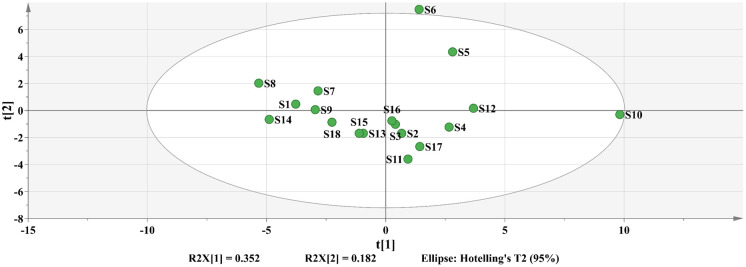
PCA analysis of S1~S18.

The S1-S18 common peak data were imported into the SIMCA software, the missing value was filled with half of the minimum value, and the component was eliminated if the missing value reached 20%. The PCA approach was used for statistically analyzing the HPLC data matrix (Fig 6). *R*. *tanguticum* grown in Gannan and Qinghai had a tendency to separate t[2], however this trend was not noticeable.

#### OPLS-DA analysis results

**Fig 7:**
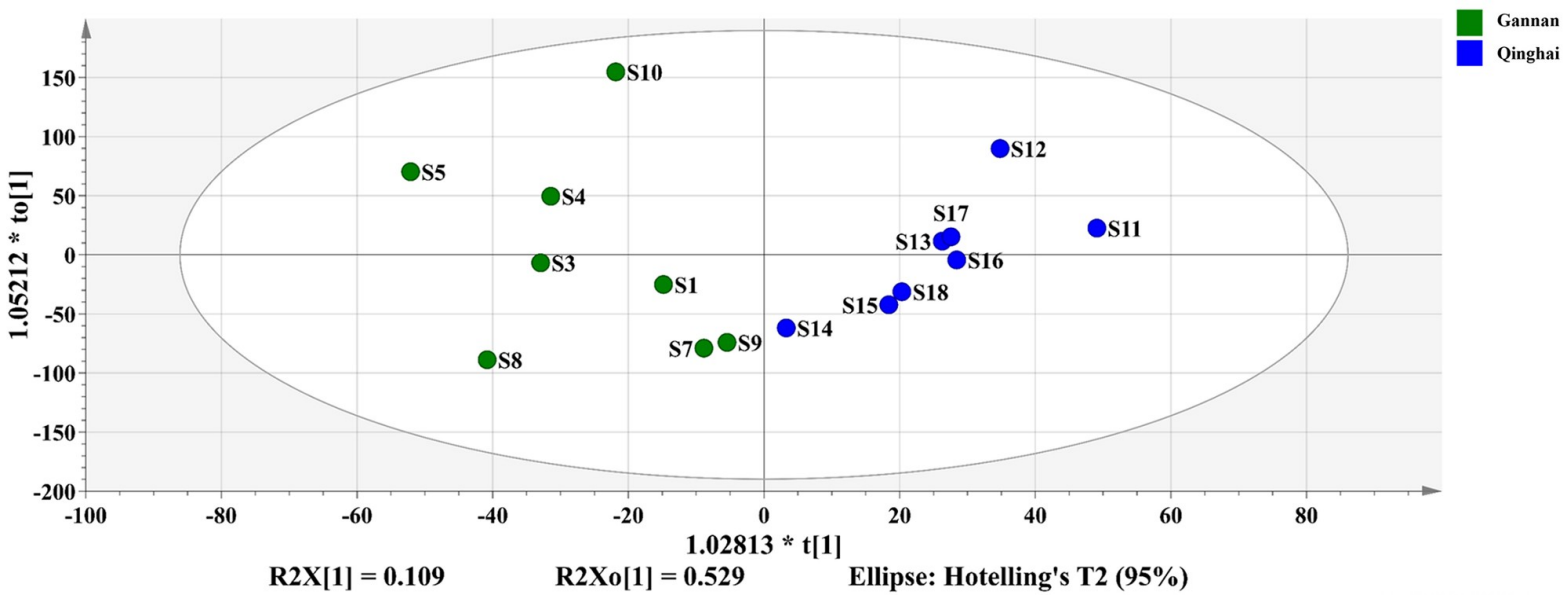
OPLS-DA.

**Fig 8:**
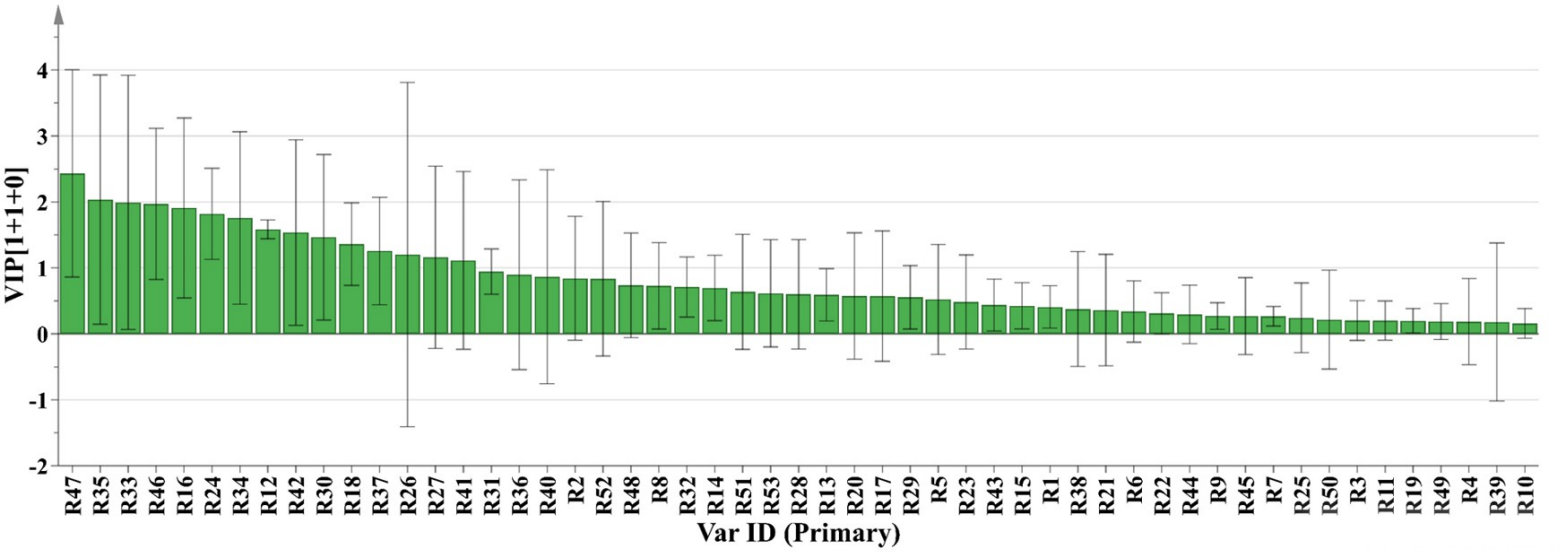
Permutations.

**Fig 9:**
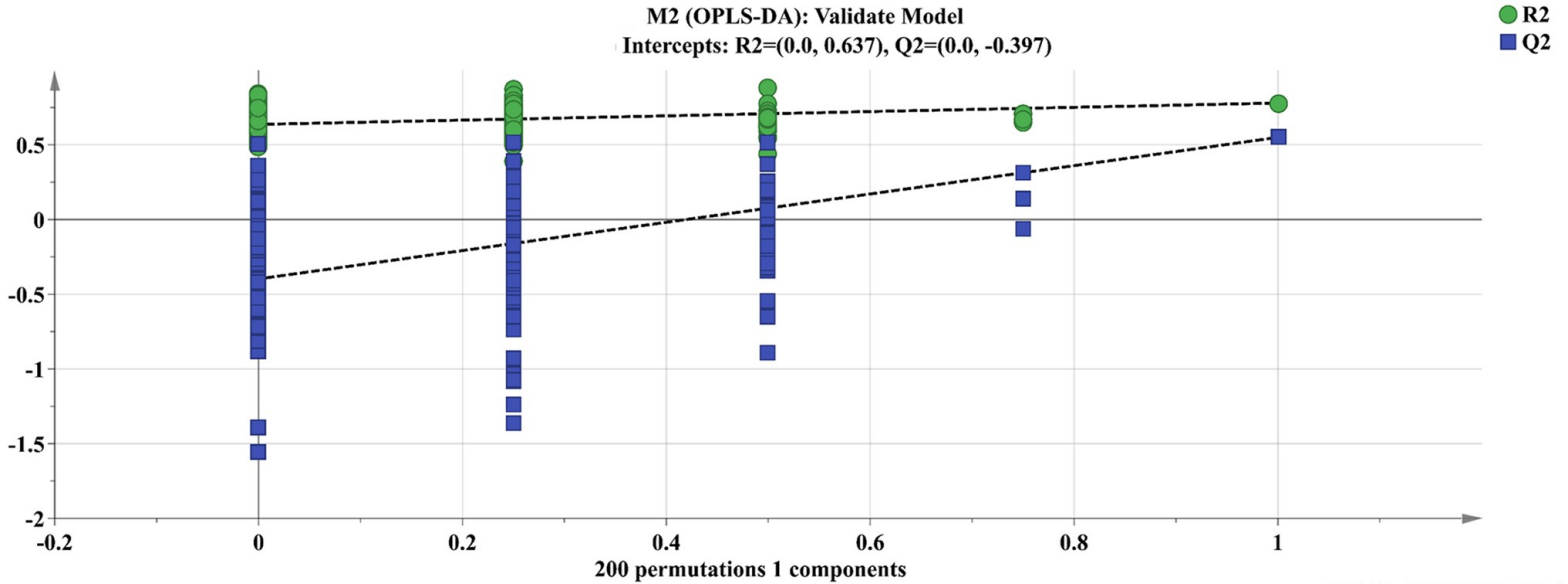
S-Plot.

**Fig 10:**
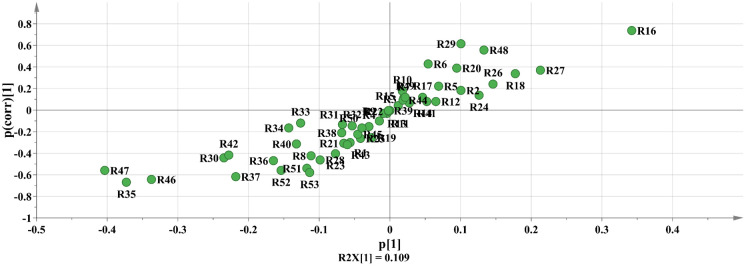
VIP Value.

According to the PCA findings, S2 (spring harvest) and S6 (outside the 95 percent confidence interval) were removed from further investigation of the component differences of *R*. *tanguticum* produced in Gannan and Qinghai. The remaining samples were separated into two groups based on their origin, Gannan and Qinghai. OPLS-DA analysis was performed between the groups, and the OPLS-DA score diagram and S-plot diagram were generated (Figs 7–9). In the OPLS-DA study, R2Y = 0.780 and Q2 = 0.550, suggesting that the two groups were well divided and that the model could predict 55.0% of the results. Permute the OPLS-DA model 200 times (Fig 10), R2 = 0.637, Q2 = -0.397, suggesting that the model is correct.

The larger was the variable importance of projection (VIP) value of the point farther from the origin of the "s" curve in the figure, the greater was the contribution to the grouping. According to the s-plot analysis, coupled with the VIP value (Fig 9). Components with a VIP ≥ 1 were eliminated, and compounds with differences were found in conjunction with P < 0.05. According to these findings, R16, R37, R46, and R47 (Aloe emodin) are the key differentiating components of Rheum tanguticum produced in Gannan and Qinghai.

### Influence of longitude, latitude and altitude on composition

Pearson’s correlation coefficient was used to examine the relationship between longitude, latitude, altitude, and composition (Table 9). Longitude was significantly positively correlated with R28 and R30 (P < 0.05), and a very significantly positively correlated with R35, R36, R37, R46, and R47 (P < 0.01). Latitude was significantly negatively correlated with R34, R35, and R40 (P < 0.05), and extremely significantly negatively correlated with R28, R30, R36, R37, R46, and R47 (P < 0.01). Altitude was significantly positive correlation with R36 and R37 (P < 0.01).

**Table 9:** Pearson correlation test table for the influence of longitude, latitude and altitude on composition.

Composition	Longitude		Latitude		Altitude	
	Correlation Coefficient	Significance	Correlation Coefficient	Significance	Correlation Coefficient	Significance
**R1**	0.291	0.241	-0.213	0.396	-0.063	0.804
**R2**	0.126	0.618	0.093	0.712	-0.333	0.178
**R3**	0.483	0.188	-0.345	0.363	0.242	0.531
**R4**	0.242	0.333	-0.314	0.204	-0.260	0.298
**R5**	0.109	0.668	-0.026	0.918	-0.311	0.209
**R6**	-0.061	0.810	0.052	0.837	-0.274	0.272
**R7**	0.275	0.285	-0.129	0.621	-0.032	0.903
**R8**	0.401	0.099	-0.342	0.164	0.188	0.455
**R9**	0.251	0.315	-0.186	0.460	0.140	0.579
**R10**	-0.115	0.660	0.200	0.443	-0.013	0.961
**R11**	0.055	0.827	-0.016	0.949	0.097	0.703
**R12**	0.233	0.353	-0.130	0.606	-0.035	0.891
**R13**	0.170	0.501	-0.202	0.421	-0.167	0.507
**R14**	0.229	0.360	-0.081	0.748	0.203	0.419
**R15**	0.079	0.754	0.081	0.748	0.108	0.671
**R16**	-0.216	0.389	0.314	0.204	-0.327	0.185
**R17**	-0.040	0.883	0.096	0.725	-0.102	0.708
**R18**	-0.011	0.966	0.087	0.732	-0.154	0.541
**R19**	0.434	0.159	-0.344	0.273	0.112	0.728
**R20**	0.093	0.732	-0.172	0.524	0.011	0.969
**R21**	0.416	0.123	-0.265	0.341	0.480	0.070
**R22**	0.045	0.869	0.036	0.896	-0.193	0.473
**R23**	0.389	0.111	-0.254	0.309	0.300	0.227
**R24**	0.135	0.594	-0.017	0.945	-0.046	0.857
**R25**	0.544	0.055	-0.414	0.160	0.272	0.368
**R26**	-0.185	0.463	0.174	0.490	-0.146	0.564
**R27**	-0.126	0.618	0.124	0.624	-0.198	0.431
**R28**	0.616*	0.025	-0.738**	0.004	0.366	0.218
**R29**	-0.491	0.217	0.540	0.167	0.123	0.772
**R30**	0.548*	0.028	-0.649**	0.007	0.341	0.196
**R31**	0.347	0.296	-0.347	0.296	0.102	0.766
**R32**	0.382	0.246	-0.382	0.246	0.066	0.847
**R33**	0.328	0.299	-0.328	0.299	0.213	0.506
**R34**	0.521	0.056	-0.595*	0.025	0.456	0.101
**R35**	0.773**	0.002	-0.617*	0.025	0.276	0.362
**R36**	0.704**	0.003	-0.704**	0.003	0.655**	0.008
**R37**	0.821**	0.002	-0.821**	0.002	0.749**	0.008
**R38**	0.574	0.106	-0.495	0.175	0.455	0.218
**R39**	0.345	0.299	-0.556	0.076	0.374	0.258
**R40**	0.585	0.059	-0.642*	0.033	0.249	0.460
**R41**	0.240	0.452	-0.166	0.605	0.122	0.706
**R42**	0.465	0.175	-0.518	0.125	0.269	0.453
**R43**	0.403	0.109	-0.458	0.065	0.468	0.058
**R44**	0.019	0.946	-0.099	0.715	0.226	0.400
**R45**	0.351	0.264	-0.307	0.332	0.373	0.233
**R46**	0.652**	0.005	-0.719**	0.001	0.307	0.231
**R47**	0.634**	0.005	-0.616**	0.006	0.407	0.093
**R48**	-0.354	0.163	0.252	0.329	-0.029	0.911
**R49**	0.373	0.232	-0.315	0.319	-0.154	0.633
**R50**	0.105	0.677	-0.097	0.703	0.155	0.538
**R51**	0.358	0.145	-0.294	0.236	0.145	0.567
**R52**	0.358	0.145	-0.266	0.286	0.201	0.424
**R53**	0.258	0.317	-0.188	0.469	0.173	0.507

*, at the level of 0.05 (two tailed), the correlation is significant;

**, At the level of 0.01 (two tailed), the correlation is significant.

## Discussion

The fingerprints of *R*. *tanguticum* are extensively researched; however, the distinctions between *R*. *tanguticum* from different production locations are still not well investigated. FTIR spectroscopy was used to examine the methanol extract of *R*. *tanguticum* from various production sites. According to cluster analysis, some discrepancies existed in the FTIR spectra from various manufacturing locations. The double index analysis sequence of the common peak and variation peak was further established, which can differentiate various production locations of medicinal materials to some extent. The alcohol extract may reduce the impact of several water-soluble components. To further investigate the relationship between components and production areas, the HPLC fingerprint of *R*. *tanguticum* was established, and chemical pattern discriminant analysis, such as PCA and OPLS-DA analysis, was introduced into the differential component analysis of *R*. *tanguticum* from different production areas. Differential components of *R*. *tanguticum* from Gannan and Qinghai were obtained for their distinction. The three elements of longitude, latitude, and altitude were preliminarily identified by evaluating the sample collection information, and the link between the different components and these three factors was built using multiple linear regression. Regression equations between the nine components and longitude and latitude were effectively established, and the link between longitude and latitude and components was discussed further.

The quality of *R*. *tanguticum* varied according to the growing environment. In terms of composition, R16 of *R*. *tanguticum* produced in Gannan was substantially lower than that produced in Qinghai (P < 0.05), whereas R37, R46, and R47 were significantly greater (P < 0.05). Longitude, latitude, and altitude all impacted the quality of medicinal products because they had an influence on the components. This can guide us in determining the origin of therapeutic ingredients and provides a theoretical basis for planting *R*. *tanguticum* in various production locations.

The quality of *R*. *tanguticum* depends on numerous factors, including temperature, humidity, precipitation, illumination time, longitude, latitude, altitude, slope, and soil characteristics. The longitude, latitude, and altitude evaluated in the experiment were merely a subset of the influencing elements, which have limits and cannot fully account for the impact of growth factors on the quality of *R*. *tanguticum*. Furthermore, human elements are critical for the widespread adoption of artificial farming [40]. Consequently, different growth environments can be included in the scope for investigating further development and utilization of *R*. *tanguticum*, and ecological factors that promote the accumulation of secondary metabolites can be fully considered to improve the quality of medicinal materials. In conclusion, the results of our study can provide insights in its quality control and aid in establishing a natural medication traceability system.

## Conclusions

The different production locations of *R*. *tanguticum* can be distinguished using FTIR and HPLC. The double index analysis sequence of common and variation peaks may differentiate distinct production locations of medicinal materials. The composition of *R*. *tanguticum* components from various sources varied substantially by the result of OPLS-DA. The key differential components of *R*. *tanguticum* produced in Gannan and Qinghai were discovered to be R16, R37, R46, and R47 (Aloe emodin) (*VIP ≥ 1 and P < 0*.*05*). Longitude, latitude, and altitude were all found to impact *R*. *tanguticum* quality by influencing the accumulation of components. Longitude was significantly positively correlated with R28 and R30 (*P < 0*.*05*), and a very significantly positively correlated with R35, R36, R37, R46, and R47 (*P < 0*.*01*). Latitude was significantly negatively correlated with R34, R35, and R40 (*P < 0*.*05*), and extremely significantly negatively correlated with R28, R30, R36, R37, R46, and R47 (*P < 0*.*01*). Altitude was significantly positive correlation with R36 and R37 (*P < 0*.*01*). The findings of this study can be used to plan planting of *R*. *tanguticum*.
